# Recent progress in stimuli‐responsive DNA‐based logic gates: Design, working principles and biological applications

**DOI:** 10.1002/smo.20230023

**Published:** 2024-02-14

**Authors:** Ling Sum Liu, Hoi Man Leung, Yuzhen Cai, Pik Kwan Lo

**Affiliations:** ^1^ Department of Chemistry and State Key Laboratory of Marine Pollution City University of Hong Kong Kowloon Tong Hong Kong SAR China; ^2^ Department of Chemistry Molecular Sciences Research Hub Imperial College London, White City Campus London UK; ^3^ Key Laboratory of Biochip Technology, Biotech and Health Care Shenzhen Research Institute of City University of Hong Kong Shenzhen China

**Keywords:** DNA logic gates, drug delivery, imaging, molecular computation, stimuli‐responsive

## Abstract

Stimuli‐responsive DNA‐based logic gates have emerged as a promising field at the intersection of synthetic biology and nanotechnology. These gates exploit the unique properties of DNA molecules to perform programmable computational operations in response to specific stimuli. This review provides a comprehensive overview of recent advancements in the design, working principles, and applications of stimuli‐responsive DNA‐based logic gates. The progress made in developing various types of logic gates triggered by metal ions, pH, oligonucleotides, small molecules, proteins, and light is highlighted. The applications of these logic gates in imaging and biosensing, drug delivery, synthetic biology and molecular computing are discussed. This review underscores the significant contributions and future prospects of stimuli‐responsive DNA‐based logic gates in advancing the field of nanotechnology.

## Graphical abstract

This review focuses on the discussion of DNA‐based logic gates that respond to different types of stimuli including metal ions, changes in pH, small molecules, DNA strands and light. The biological applications of DNA logic gates in bioimaging/diagnosis, targeted drug delivery for treatment of cancers and cellular modulation are also highlighted.

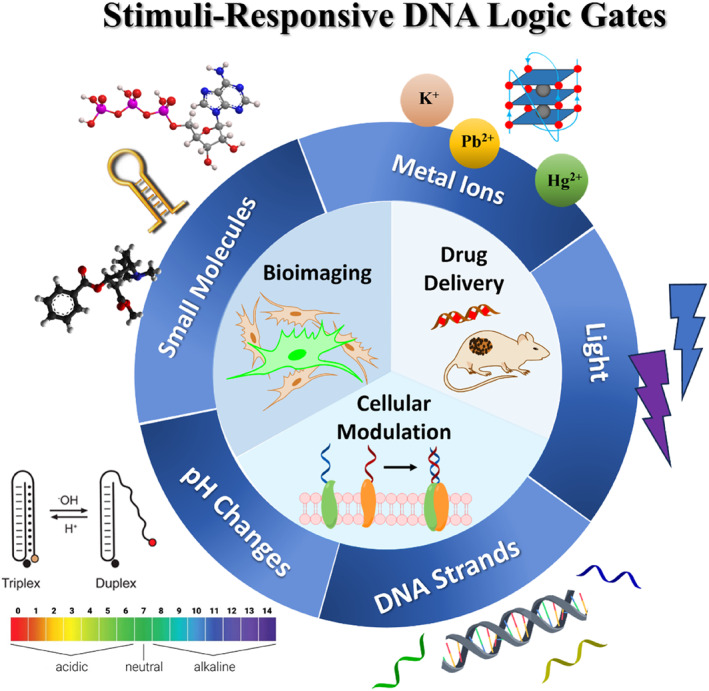

## 1 INTRODUCTION

Stimuli‐responsive DNA‐based logic gates have emerged as a captivating and rapidly evolving field at the intersection of synthetic biology, nanotechnology, and computation. These logic gates exploit the inherent programmability and versatile properties of DNA molecules to perform computational operations in a stimuli‐dependent manner. By utilizing the unique molecular recognition capabilities of DNA, researchers have made significant strides in designing sophisticated logic gates that can process and respond to specific signals or environmental cues. The ability to manipulate and engineer DNA sequences with precision has paved the way for the development of stimuli‐responsive DNA‐based logic gates. These gates offer distinct advantages over traditional electronic logic gates, such as their compatibility with biological systems, high specificity, and potential for integration into complex circuits. Moreover, DNA‐based logic gates can be tailored to respond to a wide range of stimuli.

To drive the operation of DNA logic devices, a commonly employed approach is the toehold‐mediated DNA strand displacement reaction. In this process, a specific toehold domain at one end of a double‐stranded DNA (dsDNA) binds to its complementary single‐stranded DNA (ssDNA) input, initiating the displacement reaction. The partially bound ssDNA strand then undergoes branch migration, displacing the initially bound DNA with the same sequence from the double helix strand and releasing a ssDNA as the output. The first group to employ this strategy was Winfree's group, which developed DNA‐based logic circuits capable of performing various functions such as AND, NOT, and OR gates.^[1]^ Subsequently, several other groups have designed enzyme‐free, unlabeled, or labeled fluorescent DNA logic gates using the strand displacement reaction. These gates can be employed to detect low‐abundance miRNAs, convert small molecules into logic outputs, or release model drugs.[^2^, ^3^, ^4^] The nucleobase sequences of DNA encode significant functional and structural information, enabling the rational design of stimuli‐triggered DNA logic devices. Firstly, cytosine‐rich DNA oligonucleotides fold into i‐motif structures under slightly acidic pH or in the presence of Ag^+^ ions at neutral pH. Taking advantage of this property, Shi et al. utilized aptamer AS1411 to construct OR and INHIBIT logic gates based on the formation of i‐motif structures in response to H^+^ and/or Ag^+^ inputs. The deformation of the i‐motif structure serves as the logic output.^[5]^ A turn‐on fluorescence signal from a fluorescent probe is generated once the formation of the i‐motif is recognized. Wang et al. also demonstrated enzyme‐driven i‐motif formation for NOR and NAND logic operations.^[6]^ Secondly, guanosine‐rich DNA strands fold into G‐quadruplex (G4) structures in response to ions such as K^+^ or NH_4_^+^. Wang's group developed a series of label‐free DNA logic gates based on the formation and deformation of G4 DNAzyme in response to hemin and target DNA strands, respectively.^[7]^ The logic output, which is easily observed by the naked eye, involves the generation of oxidized 3,3′,5,5′‐tetramethylbenzenidine sulfate (TMB), resulting in a blue color change. Moreover, aptamers, which are single‐stranded nucleic acids, can self‐assemble into specific high‐level structures and bind to their targets with high specificity and affinity. Adenosine, adenosine triphosphate (ATP), and thrombin‐binding aptamers have been utilized to construct DNA logic devices for molecular sensing.[^8^, ^9^]

Molecular beacon probes, introduced by Kramer's group in 1996, are single‐stranded oligonucleotides folded into stem‐loop structures with fluorophores and quenchers integrated at opposite ends.^[10]^ These folded molecular beacon (MB) probes exhibit a high response to specific sequence‐designed DNA strands, metal ligands, or biothiols (inputs), inducing a conformational change that opens the MB probe and restores the fluorescence signal as outputs.[^11^, ^12^, ^13^] Recently, Kolpashchikov demonstrated the high‐level integration of these simple MB probes into more sophisticated DNA‐based circuits for inter‐gate communication and information processing.^[14]^ Additionally, researchers have constructed nanoscale DNA logic devices based on materials such as graphite oxides,^[15]^ silica,^[16]^ gold nanoparticles (GNPs),^[17]^ or gold electrodes.^[18]^ These devices can be operated by inputs such as cysteine molecules, metal ions, proteins, or complementary DNA (cDNA) strands, and generate luminescent or electrochemical signals as outputs.

Inspired by recent advances in DNA nanotechnology, the construction of self‐assembled 2D and 3D DNA nanostructures capable of conformational or structural changes is of great importance for DNA logic circuitry. Li et al. demonstrated the use of a 2D quasi‐triangular DNA template to generate three‐input and multiple‐input Boolean logic circuits based on Toehold‐mediated strand displacement (TMSD).^[19]^ The restoration of fluorescence intensity of the fluorophore is used to recognize and report the outputs when two or more DNA strand inputs are present. Buckbout‐While et al. also employed the same displacement strategy to create reconfigurable one‐, two‐, and three‐input logic gates based on a 2D triangular cyanine‐labeled three‐arm DNA switch.^[20]^ Recently, Wang et al. and Zadegan et al. designed logic gates on DNA origami nanostructures such as rectangular‐shaped DNA origami tiles^[21]^ or 3D DNA origami box^[22]^ for microRNA (miRNA) analysis. These devices recognize different miRNAs (inputs) while the detectable Förster resonance energy transfer (FRET) signals or nanoscale symbols were given out as outputs. Fan's group also successfully built different scaffold logic devices including AND, OR, XOR, INHIBIT, and half‐adder by using a number of reconfigurable 3D DNA tetrahedra consisting of dynamic sequences which are sensitive to Hg^2+^ ions, ATP, proton and complementary nucleic acid strands (inputs).^[5]^ Signals were obtained as outputs when its structure compressed in response to external inputs.

This review article provides a comprehensive overview of the recent advancements in stimuli‐responsive DNA‐based logic gates. It aims to present representative examples and offer detailed explanations of their design and mechanisms for triggering the response of the DNA logic system to various stimuli including metal ions, pH, oligonucleotides, small molecules, proteins, and light (Table 1). The recent applications of stimuli‐responsive DNA‐based logic gates, covering imaging, biosensing, diagnostics, drug delivery systems, and molecular biocomputing will be delved into. While significant progress has been achieved, there are still challenges and limitations that require attention. The current technical hurdles and potential opportunities for improvement, emphasizing the need for robust and reliable gate designs, will be discussed. Moreover, emerging trends and future directions that hold promise for the continued development and application of stimuli‐responsive DNA‐based logic gates will also be highlighted.

**TABLE 1:** Summary of DNA logic gates responsive to different kinds of stimuli and their corresponding conformational changes.

Stimulus	Conformational change induced by	References
Metal ions	• Formation of G‐quadruplex	[23]
	• Formation of i‐motif	[24, 25, 26]
	• T‐T and C‐C base pair interactions	[27, 28, 29]
	• DNAzyme‐mediated cleavage	[30]
pH	• Formation of C‐G:C+ triplex	[31, 32]
	• Formation of i‐motif	[33]
DNA oligonucleotides	• Toehold‐mediated strand displacement (TMSD)	[34, 35, 36, 37]
	‐ Catalytic hairpin assembly (CHA)	[38, 39]
	‐ Hybridization chain reaction (HCR)	[40, 41]
	• DNAzyme‐mediated cleavage	[42, 43, 44]
Small molecules/proteins	• Binding to target molecules by DNA aptamers	[45, 46, 47, 48, 49, 50, 51, 52, 53, 54, 55, 56]
	• Antibody recognition by antigen functionalized DNA	[6, 57, 58]
Light	• Photo‐isomerization	[59]
	• Photo‐uncaging	[60, 61, 62, 63]

## 2 STIMULI‐RESPONSIVE DNA LOGIC GATES

### 2.1 By metal ions

**FIGURE 1:**
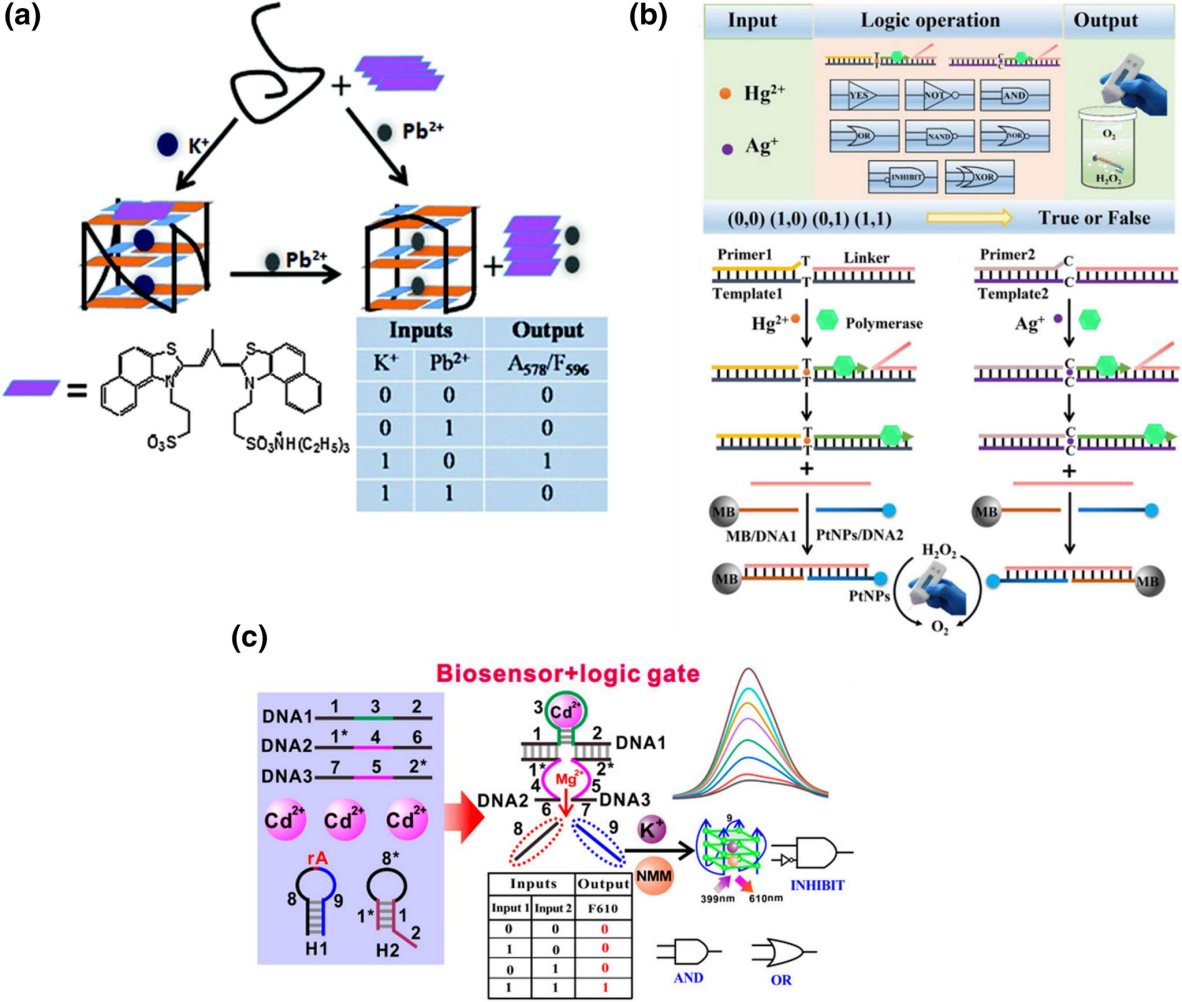
(a) Dual metal‐sensitive G‐quadruplex structures as scaffolds for logic gates. Reproduced with permission.^[23]^ Copyright © 2016, Royal Society of Chemistry. (b) DNA logic gates with the output of gas pressure for the detection of Hg^2+^ and Ag^+^ ions. Reproduced with permission.^[27]^ Copyright © 2023, American Chemical Society. (c) A DNAzyme‐mediated DNA logic system for the detection of Cd^2+^ ions. Reproduced with permission.^[30]^ Copyright © 2020, American Chemical Society.

Nucleic acids undergo folding into secondary structures through non‐covalent interactions, including ion‐dipole attractions between metal ions and nucleobases. One example is the formation of a G4, where guanine (G)‐rich oligonucleotides fold into a tetrad structure, adopting a helical geometry and stabilizing through central metal ions.^[64]^ Due to their reliance on metal ions, the addition of metal ions can serve as inputs for logic gates to induce G4 formation. Chen et al. demonstrated an INHIBIT gate by utilizing G4s as a scaffold and two metal ions as inputs (Figure 1a).^[23]^ They employed a G‐rich DNA strand capable of forming different G4 structures upon the addition of K^+^ or Pb^2+^ ions, and a fluorescent G4‐interactive ligand as the output signal. Specifically, the K^+^ ion induced the G‐rich DNA strand to fold into a parallel G4 motif, while the Pb^2+^ ion induced the G‐rich DNA strand to fold into an antiparallel G4 motif. The fluorescent G4‐interactive ligand selectively bound to the parallel G4 motif, resulting in a strong emission signal in the presence of K^+^ ions, representing a positive output. Conversely, the antiparallel G4 motif induced by Pb^2+^ ions exhibited weak interaction with the G4‐interactive ligand, leading to a weak fluorescent signal as a negative output.

Another secondary structure, known as the intercalated‐motif (i‐motif), is formed by folding cytosine‐rich oligonucleotides into a quadruplex structure, typically under acidic conditions.^[65]^ It was later discovered that the i‐motif can also be formed in the presence of Ag^+^ ions.^[24]^ Shi et al. exploited an OR logic gate by utilizing a DNA strand sensitive to H^+^ and Ag^+^ ions as the scaffold, with a fluorescent dye binding to the i‐motif as the output signal.^[25]^ In this setup, the H^+^ and Ag^+^ ions acted as inputs, inducing the formation of the i‐motif structure, and the dye bound to the i‐motif, resulting in the release of the output signal.

In addition to facilitating the formation of these secondary structures, metal ions can provide an attractive force to anneal two DNA strands. Hg^2+^ and Ag^+^ ions can bridge thymine (T) and cytosine (C), respectively, and serve as inputs to trigger logic gate operations. Rana et al. employed this strategy to assemble AND and OR logic gates based on a dual metal ion‐responsive platform.^[28]^ The logic gate scaffold was constructed using GNPs and a T‐ and C‐rich DNA single strand. Upon the addition of Hg^2+^ or Ag^+^ ions, the free T‐ and C‐rich DNA single strands were attracted by the ion‐dipole attractions and annealed, resulting in the formation of DNA double strands. The annealed DNA double strands further triggered a hybridization chain reaction (HCR), leading to a reduction in relative absorbance and ultimately providing the outputs. Annealing of DNA strands by metal ions was also utilized by Zhang et al. to construct AND, NAND, and NOR logic gates.^[29]^ They built the logic gates by assembling T‐ and C‐rich DNA single strands on a gold electrode surface as the scaffold. Upon the addition of Hg^2+^ or Ag^+^ ions, the free DNA single strands conjugated with ferrocenecarboxylic acid (Fc) were annealed, resulting in the generation of electric currents as outputs.

Recently, metal ion‐responsive DNA logic gates have been applied as detection platforms for heavy metals, which pose toxicity risks to organisms. These logic gates utilize metal ions as inputs, with the outputs providing signals for quantifying the presence of these metal ions. Lan and colleagues developed an i‐motif‐based INHIBIT logic gate that responded to Ag^+^ ions.^[26]^ In the presence of Ag^+^ ions, the i‐motif structure was induced, resulting in luminescence signals as outputs. The concentration of Ag^+^ ions could be quantified by measuring the emission intensity. This approach was successfully applied to analyze biological samples such as human serum and fishes, as well as environmental samples like lake water and soil. Shi and colleagues assembled logic gates and utilized these platforms for the detection of Hg^2+^ and Ag^+^ ions (Figure 1b).^[27]^ They took advantage of Hg^2+^ and Ag^+^ ions bridging T and C bases respectively and used DNA fragments as the scaffold for the logic gates. Specifically, these DNA fragments acted as primers, and the extension reactions were catalyzed by polymerase. In the presence of Hg^2+^ and Ag^+^ ions, the DNA fragments annealed and initiated the polymerization process. The polymerized DNA destabilized a linker strand, which connected two DNA strands bonded to magnetic beads and platinum nanoparticles (PtNPs), respectively. The aggregation of magnetic beads and PtNPs generated hydrogen peroxide, which rapidly decomposed into oxygen gas. In summary, the presence of Hg^2+^ and Ag^+^ ions served as inputs and targets for detection, while the pressure of oxygen gas served as the output, allowing for the quantification of these metal ions.

Chen and colleagues demonstrated the use of various logic gates (IMPLICATION, NOR, NAND, etc.) as detection tools for Cd^2+^ ions (Figure 1c).^[30]^ They took advantage of the inducement of DNA hairpins by Cd^2+^ ions to construct these logic gates. Specifically, the scaffolds of the logic gates consisted of inactive deoxyribozymes (DNAzymes) capable of catalyzing cleavage reactions to produce G4 DNA strands. The DNAzymes were activated upon the addition of Cd^2+^ ions, which served as both inputs and targets for detection. In the presence of the fluorescent dye N‐methyl mesoporphyrin IX (NMM), a selective G4 binding dye, the generated G4 DNA strands produced fluorescence signals. Hence, the fluorescent signal resulting from the interaction between G4s and NMM served as the output, enabling quantification of Cd^2+^ ions.

### 2.2 By pH changes

**FIGURE 2:**
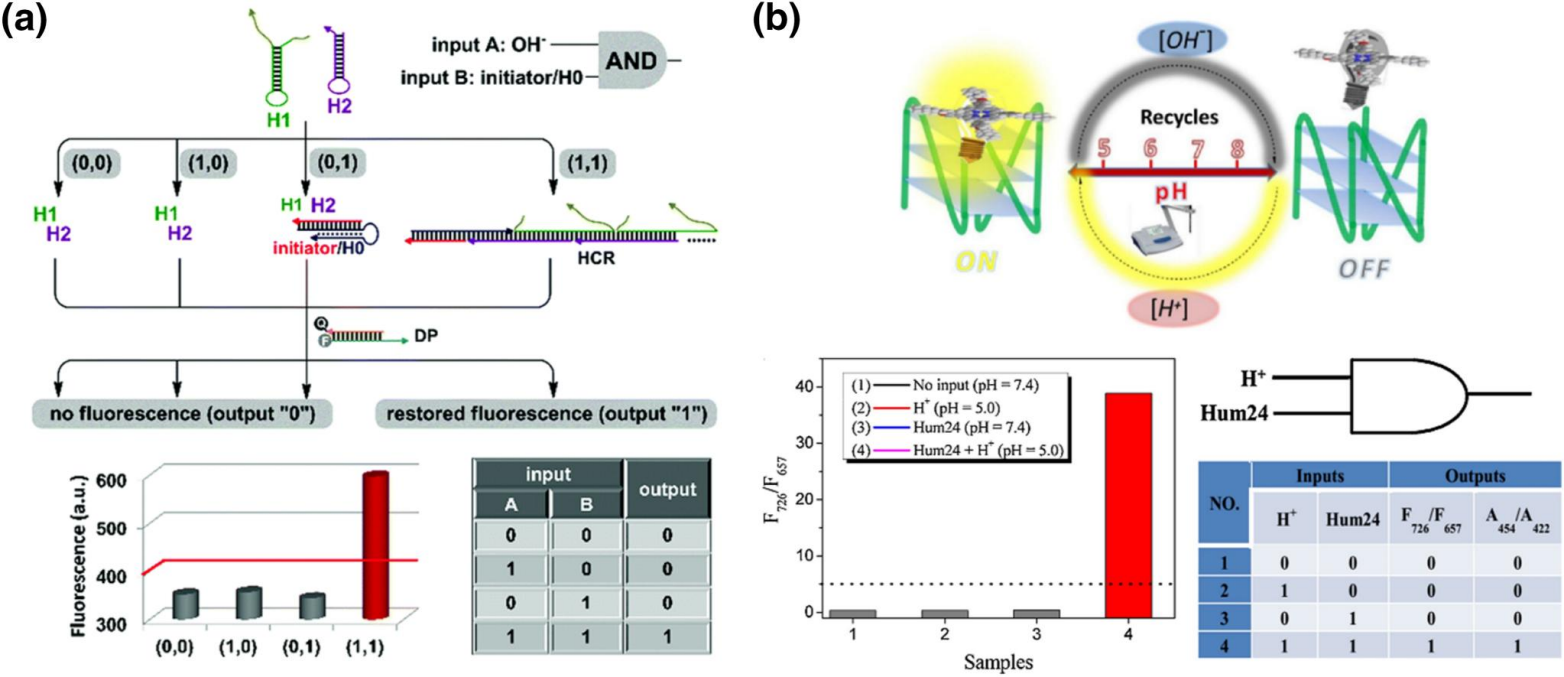
(a) AND gate execution based on a pH‐dependent DNA triplex‐helix switch. Reproduced with permission.^[31]^ Copyright © 2018, Royal Society of Chemistry. (b) DNA logic gates with AND operations utilizing protons as one of the inputs, the porphyrin derivative responded to G‐quadruplex structure in an acidic environment to give enhanced fluorescence signal. Reproduced with permission.^[67]^ Copyright © 2016, Elsevier.

The DNA triple helix is a non‐canonical secondary structure formed by a DNA strand binding to the major groove of a DNA duplex through Hoogsteen hydrogen bonding. The most common type of DNA triplex is the C‐G:C^+^ pairing, driven by the electrostatic attraction between protonated cytosine at *N‐3* and the lone pair electrons on nucleobases.^[66]^ Consequently, the addition of protons can serve as inputs for logic gates, facilitating the construction of DNA triple helices, enabling the completion of logic gate systems, and generating the corresponding outputs. Qi and colleagues leveraged the protonation and deprotonation processes involved in DNA triplex formation to develop an AND logic gate (Figure 2a).^[31]^ Specifically, the addition or removal of protons acted as inputs for the logic gate. The differential formation of DNA duplexes or triplexes guided subsequent chain reactions, resulting in the production of distinct DNA strands as outputs. Subsequently, Zhang's group also utilized the same strategy to exploit the AND and OR logic gates.^[32]^ They employed the formation of triplexes and duplexes through the addition of proton or hydroxide ions as inputs, with the resulting DNA complexes serving as outputs. Due to the difference in mobility between DNA triplexes and duplexes, the outputs were easily distinguishable in gel electrophoresis results.

pH‐sensitive DNA logic gates have been applied as pH sensing platforms, utilizing protons as inputs and fluorescent dyes selective toward DNA secondary structures as outputs. Pu et al. and Huo et al. demonstrated DNA logic gates that responsively detected protons as pH detection platforms.[^33^, ^67^] Specifically, the scaffolds of these logic gates consisted of G‐ or C‐rich DNA strands capable of folding into G4 or i‐motif structures under acidic conditions. In the presence of protons, which served as inputs for the logic gates, the DNA secondary structures were induced (Figure 2b). The fluorescent macromolecules synthesized in‐house, which selectively bound to DNA secondary structures, generated luminescence signals as outputs, providing an analogy to pH levels.

### 2.3 By DNA oligonucleotides

Toehold‐mediated strand displacement is a widely used technique in DNA nanodevices that exhibit dynamic functionalities. The reaction mechanism involves hybridizing an invading ssDNA to the toehold domain of a partially double‐stranded complex. This is followed by branch migration through a recognition domain, resulting in the formation of a more stable duplex.^[68]^ Reversibly, the newly formed complex leaves a toehold domain available for further displacement reactions. The TMSD reaction is highly specific to the sequence of the oligonucleotides, and its kinetics can be precisely controlled by the base sequence and length of the toehold region. With its advantages of high programmability, modularity, and specificity, the TMSD reaction serves as a promising tool for constructing DNA logic devices.

Over the past few decades, elementary two‐input DNA logic operations, such as OR, AND, NOR, NAND, and XNOR, have been successfully demonstrated in TMSD‐based systems using designated oligonucleotide inputs.[^1^, ^69^, ^70^] These achievements have driven the development of DNA circuits for more complex computations. Multiple concurrent contrary logic gates, including INHIBIT/IMPLICATION, XOR/XNOR, and MAJORITY/MINORITY, can be implemented using DNA hairpin‐templated silver nanoclusters (AgNCs). The opening of the hairpin structure is controlled by strand displacement upon the addition of input oligonucleotides, resulting in emission changes of AgNCs at 565 and 630 nm.^[34]^ Li et al. constructed XOR and AND gates using multiple DNA duplexes, where single‐stranded DNAs triggered successive displacement reactions.^[35]^ By integrating the XOR and AND gates, they built a half‐adder logic circuit with two fluorescent outputs (FAM and HEX). Xie et al. designed a compact yet efficient architecture for a full‐adder circuit.^[36]^ They utilized three XOR‐AND double‐logic gates in parallel to perform the full adder function using only 13 DNA strands. The XOR and AND logic were computed based on cooperative strand displacement reactions, enabling 4‐bit and 6‐bit computations. Fan's group developed DNA switching circuits that realized cascade displacement reactions, allowing for the implementation of full‐adder and complex square root functions.^[37]^

**FIGURE 3:**
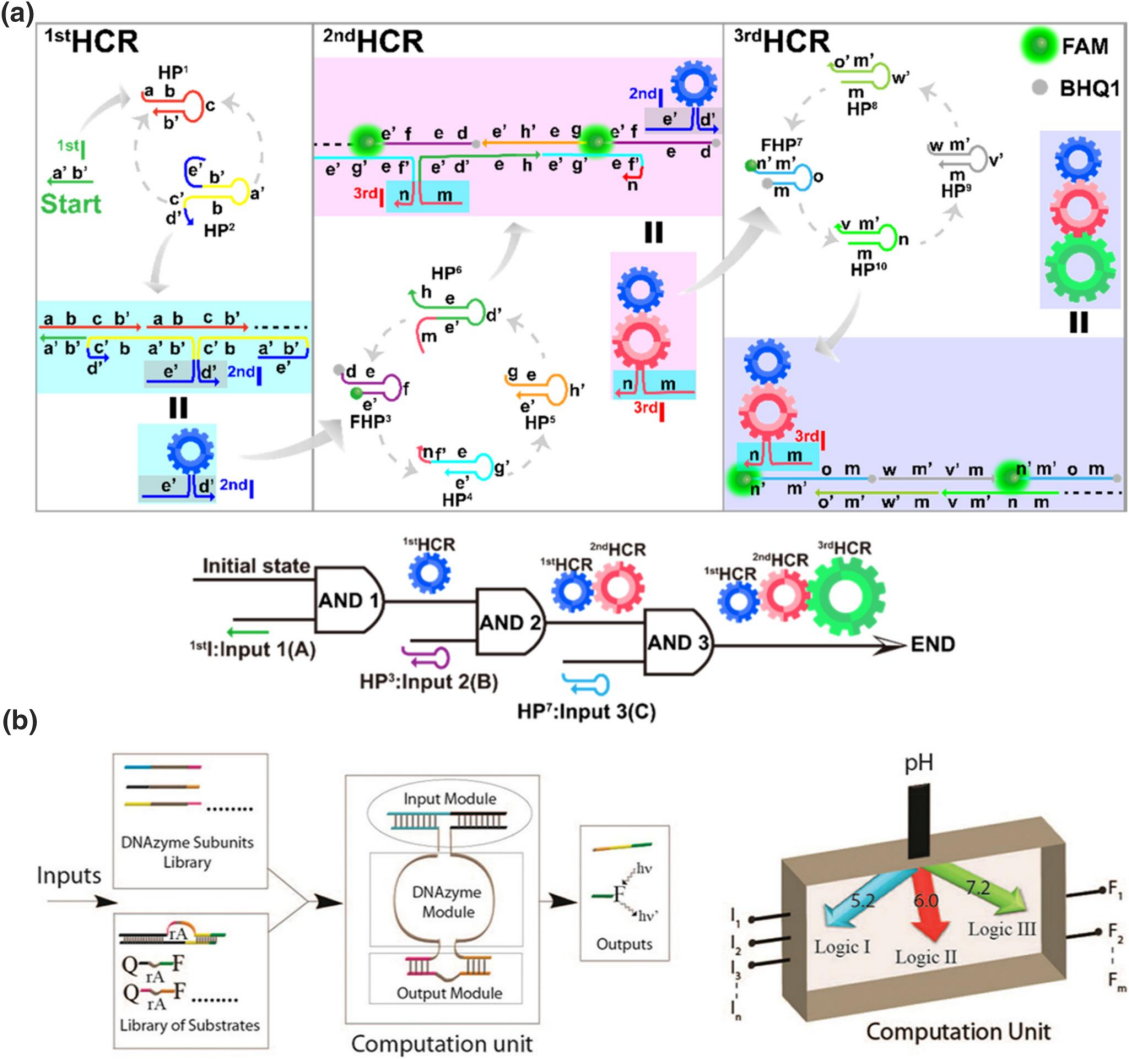
(a) Implementation of a three‐layer hierarchical DNA logic system using hybridization chain reaction. Reproduced with permission.^[41]^ Copyright © 2021, American Chemical Society. (b) Molecular logic gates constructed by Mg^2+^‐dependent DNAzyme. Reproduced with permission.^[42]^ Copyright © 2012, American Chemical Society.

Catalytic hairpin assembly (CHA) and HCR are subcategories of TMSD that find extensive applications for signal amplification in DNA logic operations. In 2008, Yin et al. discovered that the assembly of two DNA hairpins into a duplex structure could be initiated by an initiator strand.^[71]^ The initiator strand opened a DNA hairpin through TMSD, resulting in a partially hybridized duplex. Subsequently, the second DNA hairpin displaced the initiator strand, forming a more stable duplex. The displaced initiator strand acted as a catalyst, initiating another cycle of hairpin assembly. Li et al. integrated CHA with a double‐crossover (DX) motif‐based nanotweezer to implement AND functions.^[38]^ The DX tweezer transitioned from an open to a closed state upon the input of two microRNAs (miRNA‐21 and miRNA‐155), leading to the formation of a proximity‐dependent DNAzyme for signal reporting. Xu's group demonstrated a miRNA‐CHA three‐input concatenated logic system.^[39]^ Fluorophore‐labeled DNA hairpins were immobilized on a magnetic bead, and miRNA‐21, miRNA‐155, and miRNA‐Let‐7a, along with additional DNA hairpins, served as inputs to initiate CHA and generate fluorescent output signals. Catalytic hairpin assembly offers sensitive and robust signal amplification with minimal target inputs. Hybridization chain reaction operates on a similar principle as CHA, but the hairpin monomers polymerize to form a long DNA duplex.^[72]^ Chatterjee et al. constructed a DNA circuit with a domino architecture by designing a series of hairpins on the surface of a DNA origami.^[40]^ Hairpin polymerization occurred by introducing initiator strands and fuel DNA hairpins along the origami via HCR. Finally, the output hairpin at the destination displaced the quencher‐labeled strand in the reporter duplex, resulting in fluorescence recovery. Lv et al. developed a three‐layer hierarchical HCR system composed of cascaded AND gates, enabling an ultrasensitive response to trace amounts of input (Figure 3a).^[41]^ Without the first initiator strand (1stI), the HCR between three designed DNA hairpins was prohibited. The introduction of 1stI triggered the first HCR, producing the first layer of a long DNA duplex trunk. This trunk contained a tandem rearranged initiator that activated the second HCR, forming the second layer of DNA nanowires with repetitively reconstructed initiators. The repetitive reconstructed initiator entered the third HCR to generate the third layer of DNA duplex structure. This three‐layer hierarchical HCR system achieved the detection of 1stI down to 50 pM and exhibited excellent discrimination against various concentrations of 1stI when the concentration was below 1 nM.

Deoxyribozymes (DNAzymes) are biocatalysts primarily composed of DNA oligonucleotides.^[73]^ Certain DNAzymes exhibit catalytic activity solely in the presence of metal ions. Willner's group extensively developed molecular logic gates based on Mg^2+^‐dependent DNAzymes (Figure 3b).[^42^, ^43^, ^44^] They constructed various logic gates by incorporating Mg^2+^ binding cores as computational units. Specifically, DNA oligonucleotides responsive to Mg^2+^ were employed as inputs and bound to logic gate scaffolds to form complete operational machinery. Upon the addition of DNA oligonucleotides and activation of DNAzymes, specific‐catalyzed chemical reactions occurred, and the resulting products served as outputs.

### 2.4 By small molecules/proteins

**FIGURE 4:**
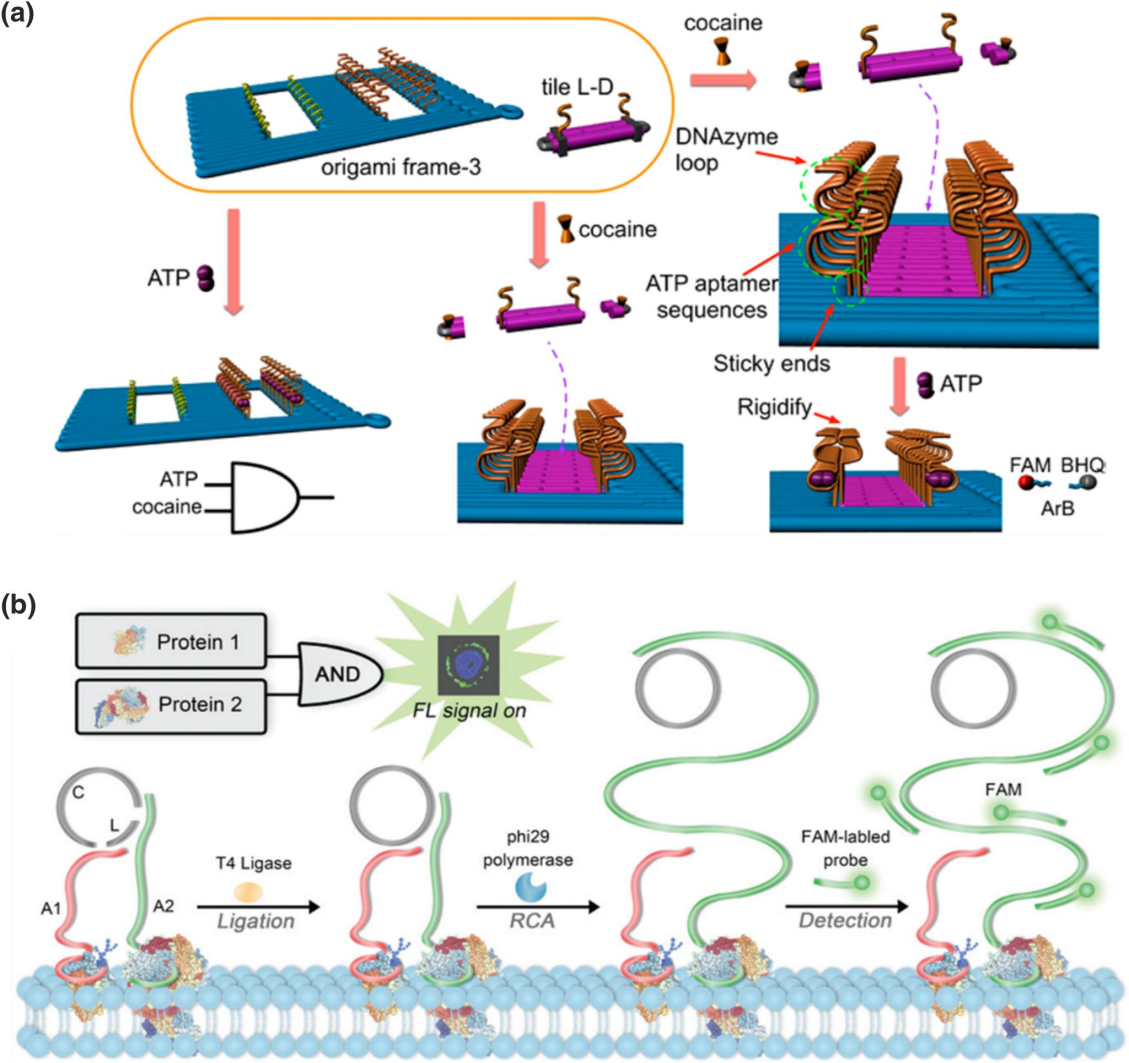
(a) Operations of the AND gate by an assembly of DNA origami using adenosine triphosphate and cocaine as inputs for activation. Reproduced with permission.^[45]^ Copyright © 2016, American Chemical Society. (b) Dual recognition of cell membrane receptors by DNA logic gate with rolling circle amplification to amplify the output signal. Reproduced with permission.^[46]^ Copyright © 2020, Royal Society of Chemistry.

DNA aptamers are short oligonucleotides that exhibit strong molecular recognition toward a wide range of small molecules or proteins.[^74^, ^75^] They can induce structural switching, making them valuable for the activation of DNA logic devices using target molecules. Zhang's group introduced an aptamer‐substrate strategy combined with the DNAzyme cleavage to control the patterning of DNA origami in a logical manner (Figure 4a).^[45]^ Initially, cocaine was added to bind with the target aptamer in a DNA tile, activating the DNAzyme cleavage to unlock the DNA tile and allow for the filling of a pre‐designed origami cavity. Subsequently, the addition of adenosine triphosphate (ATP) rigidified the DNAzyme structure, leading to the cleavage of a reporter strand conjugated with a fluorophore‐quencher pair (ArB). An AND gate configuration resulted in a significant enhancement of fluorescence. Aptamers have also been employed in the work of Liu et al. to establish an OR gate and an INHIBIT gate for sensing kanamycin and oxytetracycline.^[47]^ In this approach, a probe strand with two aptamer units was immobilized onto a magnetic bead, while a reporter strand with a G4 sequence hybridized with the probe strand. Upon introducing kanamycin and oxytetracycline, the probe strand interacted with the antibiotics, causing its disassembly and the subsequent release of the signal strand. The signal strand folded upon adding hemin, generating a chronopotentiometric signal through the H_2_O_2_‐mediated oxidation of 3,3′,5,5′‐tetramethylbenzidine by the G4/hemin DNAzyme. In the operation of an XOR logic gate developed by the group of Wang, an adenosine‐binding aptamer conjugated to GNPs played a crucial role.^[48]^ The folding of the aptamer in response to adenosine resulted in the removal of the hybridized complementary strand, bringing the conjugated dye close to the GNPs. Quenched fluorescence was detected using total internal reflection fluorescence microscopy, with this variation serving as the readout for XOR operations. Su et al. demonstrated another XOR gate based on ATP and its aptamer.^[49]^ A gold electrode surface was fabricated with methylene blue‐labeled DNA hairpins that possessed a complementary sequence to the ATP aptamer. Some of the DNA hairpins hybridized with the ATP aptamer, forming duplex structures, while others remained in a hairpin conformation. The switching between hairpin and duplex structures depended on the presence of ATP and ATP aptamer. The increased proportion of hairpin/duplex structures in response to the addition of ATP or ATP aptamer controlled the distance between methylene blue and the electrode surface, influencing electron transfer and resulting in a change in the electrochemical signal. When both inputs were present, no signal change was detected due to the unchanged proportion of hairpin/duplex structures on the electrode. Wang's team employed an anti‐bisphenol A (BPA) aptamer with bi‐affinity toward BPA and its derivative bisphenol S (BPS) to construct a three‐input two‐level logic network.^[50]^ BPA, BPS, and the anti‐BPA aptamer served as the three inputs, influencing the aggregation of GNPs. The output signals were reported by the absorption ratio (A660/A520) and visual observations.

The specific recognition capabilities of DNA aptamers also enable proteins to act as inputs and trigger logic operations through DNA‐protein interactions. For instance, Su et al. utilized a designed aptamer to recognize thrombin in a molybdenum disulfide‐based electrochemical logic sensor.^[51]^ Upon binding with thrombin, the aptamer underwent a structural change that caused the methylene blue conjugated to the aptamer to move away from the electrode, resulting in a decrease in the signal. By combining the thrombin aptamer with an ATP aptamer, an AND gate with inputs of thrombin and ATP was created. Moreover, Sun and co‐workers employed thrombin and ATP aptamers in the construction of a half‐adder and a half‐subtractor. The aptamers labeled with fluorescent quantum dots were adsorbed on graphene oxide (GO). The addition of thrombin and ATP led to the binding of the aptamers with the corresponding inputs, causing the complexes to detach from GO and resulting in enhanced fluorescence. By controlling the input of thrombin, ATP, and their respective aptamers, the fluorescence at 506 and 571 nm served as the outputs for the half‐adder and half‐subtractor operations.^[52]^

Aptamers obtained through cell‐based systematic evolution of ligands by exponential enrichment (SELEX) processes can specifically interact with membrane proteins,^[53]^ facilitating the development of logic devices for accurate diagnosis and targeted therapy. You et al. combined the aptamer recognition property with TMSD reactions to perform logic operations on cell membranes.^[54]^ Aptamers tagged with invading strands were anchored to the protein receptors, and the invading strands displaced the reporting DNA duplex conjugated with a dye or therapeutic agent. This strategy allowed for the incorporation of multiple receptors as inputs to build a cascade reaction platform, enabling a series of aptamer‐driven logical operations. Rolling circle amplification (RCA), an alternative signal amplification method to TMSD reactions, was also employed. After recognizing two proximate target protein receptors through dual aptamers, RCA was triggered by primers connected to the aptamers and a cyclized DNA template, in the presence of DNA polymerase and dNTPs. The addition of fluorophore‐conjugated DNA probes hybridized with the RCA product, ultimately reported the output signal (Figure 4b).[^46^, ^55^] In aptamer‐assisted logic operations, another signal amplification strategy utilized cyclic enzymatic signal amplification. Yu et al. employed DNA hairpins with aptamers (sgc8c and A10–3.2) to bind to the tyrosine kinase‐like 7 (PTK7) and prostate‐specific membrane antigen on the extracellular vesicle (EV) membrane.^[56]^ The DNA hairpins opened and released the hybridization region for other input hairpins, forming duplex structures. A restriction endonuclease, Nb.BbvCI, cleaved the duplex by recognizing the sequence 5′‐GCTGAGG‐3′. The cleaved fragments were captured by a DNA‐functionalized gold electrode. When K^+^ ions and hemin were introduced, the captured DNA fragments folded into a G4 structure, resulting in an electrochemical signal.

In addition to aptamer‐protein binding, the functionalization of DNA with antigens allows for specific interactions between DNA and proteins through antibody‐antigen recognition, leading to conformational changes in DNA. For instance, a tailed DNA molecular beacon was partially hybridized with complementary DNAs conjugated with digoxigenin (DIG) and dinitrophenol (DNP). The stem‐loop structure of the beacon remained closed until both anti‐DIG and anti‐DNP antibodies were introduced, causing the structure to open due to steric strain. The readout signal of the AND gate was obtained from the enhanced fluorescence emission of the fluorophore‐quencher pair conjugated to the stem region.^[57]^ This antigen recognition‐based AND gate design was also implemented in the structural changes of DNA origami. An icosahedral DNA origami containing anti‐DIG and anti‐DNP antibodies was constructed by incorporating targeting ligands at the edges of the origami. The icosahedral origami only disassembled into triangular monomers when both DIG and DNP were added, completely displacing the antibodies on the origami and triggering reconfiguration.^[58]^ Enzyme‐driven reactions provide an alternative approach to incorporate proteins into DNA‐based logic operations. Enzymes can selectively catalyze reactions with specific substrates, making them suitable for application in Boolean logic operations with specific inputs. In a study by Wang et al., acetylcholinesterase (AChE) and glucose oxidase (GOx) were used to control the hydrolysis of acetylcholine and the oxidation of glucose, resulting in the production of acidic species that stabilized the formation of DNA i‐motifs.^[6]^ The proximity of the dye and quencher in the i‐motif led to quenched fluorescence, serving as the readout for a NOR gate. AChE was replaced with invertase (INV) in a NAND gate configuration, where INV hydrolyzed sucrose instead. Only when both INV and GOx were present, gluconic acid generated from the hydrolysis of sucrose, creating a low pH environment for i‐motif formation.

### 2.5 By light

Light is a powerful tool for controlling DNA logic gates due to its non‐invasive nature, orthogonality, and high spatiotemporal accuracy. These characteristics enable the manipulation of molecular processes using different wavelengths of light as inputs. Photo‐isomerization, photochromism, and photo‐uncaging are well‐established methods for operating DNA logic gates.

Song et al. developed logic operations by utilizing photo‐isomerized DNA strands as motors to control TMSD.^[59]^ They employed azobenzene, which can transition between cis‐ and trans‐isomers upon exposure to ultraviolet (UV) and visible (Vis) light, to drive the logic gate machinery. Specifically, the azobenzene molecule existed in the cis‐form under UV light and the trans‐form under Vis light. The researchers designed and assembled the DNA scaffold in the stable trans‐form and the unstable cis‐form. UV light was used as an input to trigger TMSD and de‐hybridize the DNA strands, leading to amplification. The concentration of DNA strands was then increased and quantified as the output. An important feature of this logic gate design was its ability to reset to the initial state by irradiating it with Vis light, making the logic gate operation renewable.

Photo‐uncaging is a widely used method for manipulating DNA logic gates. Deiters's group demonstrated NAND and NOR logic gates using photo‐caged thymidine nucleotides.^[60]^ Specifically, they employed a photolabile protecting group called 6‐nitropiperonyloxymethylene (NPOM) to mask the N‐3 hydrogen bond donor on thymine. Upon UV light irradiation, the NPOM group was removed, allowing Watson‐Crick base pairing to resume. This served as the input for the logic gates and initiated strand displacement. A DNA strand conjugated with a quencher was de‐hybridized from another DNA strand conjugated with a fluorescent dye. As a result, the luminescence intensity was restored, serving as the output. Booth's group utilized a dual‐wavelength sensitive *β*‐galactosidase to implement an AND gate for cell‐free protein expression.^[61]^ They employed a coumarin photocage, which exhibited favorable photo‐uncaging kinetics and dynamics under blue light, to assemble the logic gate. Another photocage was only active under UV light. Upon irradiation with both UV and blue light (the positive inputs of the AND gate), protein expression was activated. This activation led to the catalysis of the non‐fluorescent carboxyumbelliferyl‐β‐D‐galactopyranoside (CUG) by *β*‐galactosidase, resulting in the production of fluorescent umbelliferone‐3‐carboxylic acid (UCA). Ultimately, the fluorescent signal served as the output of this AND gate.

**FIGURE 5:**
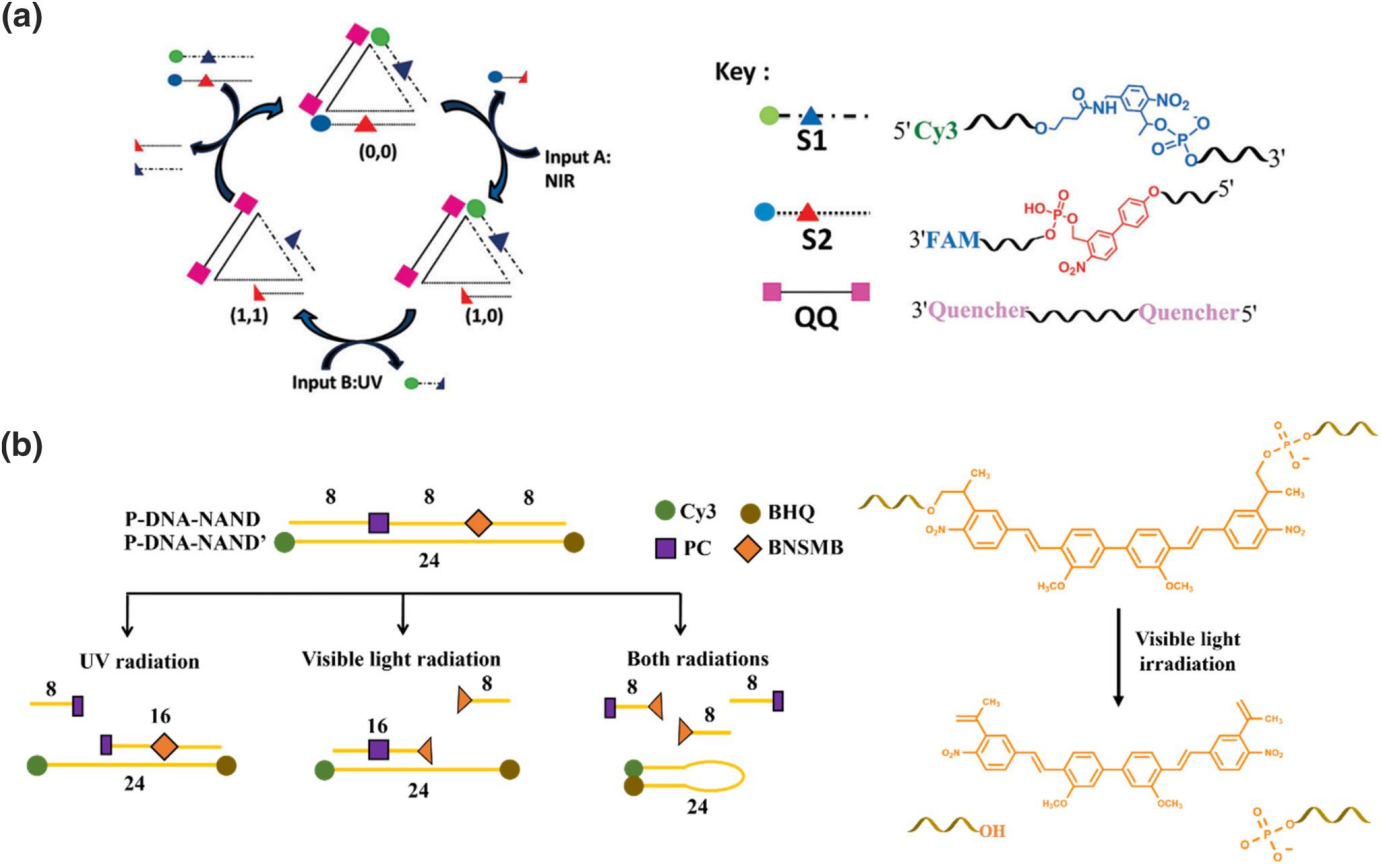
(a) Double‐stranded DNA triangle as a scaffold of OR logic gate and UV and near‐infrared lights serving as the input signals. Reproduced with permission.^[62]^ Copyright © 2015, John Wiley and Sons. (b) UV‐ and visible‐light‐responsive DNA logic gates based on the transformation of duplex to hairpin structure which altered the proximity of dye and quencher to create the output signal. The photocleavage of BNSMB is shown on the right panel. Reproduced with permission.^[63]^ Copyright © 2023, American Chemical Society.

Our research group has successfully utilized a photo‐uncaging strategy to develop two light‐responsive DNA logic gates. These gates incorporate photocleavable molecules as monomeric units within DNA oligonucleotides. In one study, an OR gate was constructed using a dsDNA triangle with various dyes and photocleavable molecules (Figure 5a).^[62]^ Specifically, 4‐nitro‐4′‐phenoxy‐1,1′‐biphenyl (4‐NB) and 2‐nitrophenyl (2‐NP) molecules were incorporated into DNA strands. 4‐NB was responsive to near‐infrared light, while 2‐NP was activated by UV light. Near‐infrared and UV irradiation served as the inputs for the logic operation. Upon irradiation, the DNA strands were cleaved, leading to the destabilization of the duplexes. This fragmentation separated the dye‐conjugated DNA strands from the quenchers, resulting in the restoration of fluorescent signals, which acted as the outputs. Subsequently, we expanded our investigations to include extensive DNA logic gates, such as AND, OR, NOR, and NAND gates, using a similar strategy with a more efficient photocleavable molecule.^[63]^ We employed 4,4′‐bis‐{8‐[4‐nitro‐3‐(2‐propyl)‐styryl]}‐3,3′‐di‐methoxybiphenyl (BNSMB) as a bi‐terminal photocleavable molecule, which exhibited improved photo‐uncaging kinetics and dynamics under visible (Vis) light. UV and Vis lights were utilized as inputs to construct the logic gates. This was achieved by annealing a DNA strand containing both BNSMB and 2‐NP with a cDNA strand carrying various dyes and quenchers. Upon UV or Vis irradiation, specific DNA fragments were destabilized, liberating the DNA strand with the dye and quencher. We took advantage of hairpin formation to generate output signals (Figure 5b). For example, in the design of a NAND gate, photocleavage of both photocleavable units destabilized the duplex, causing the release of the DNA strand with the dye and quencher. This DNA strand was designed to form a hairpin structure, bringing the dye and quencher in close proximity. The resulting diminished fluorescence signal served as the output in the logic operation.

## 3 BIOLOGICAL APPLICATIONS OF STIMULI‐RESPONSIVE DNA‐BASED LOGIC DEVICES

### 3.1 Imaging and diagnosis

**FIGURE 6:**
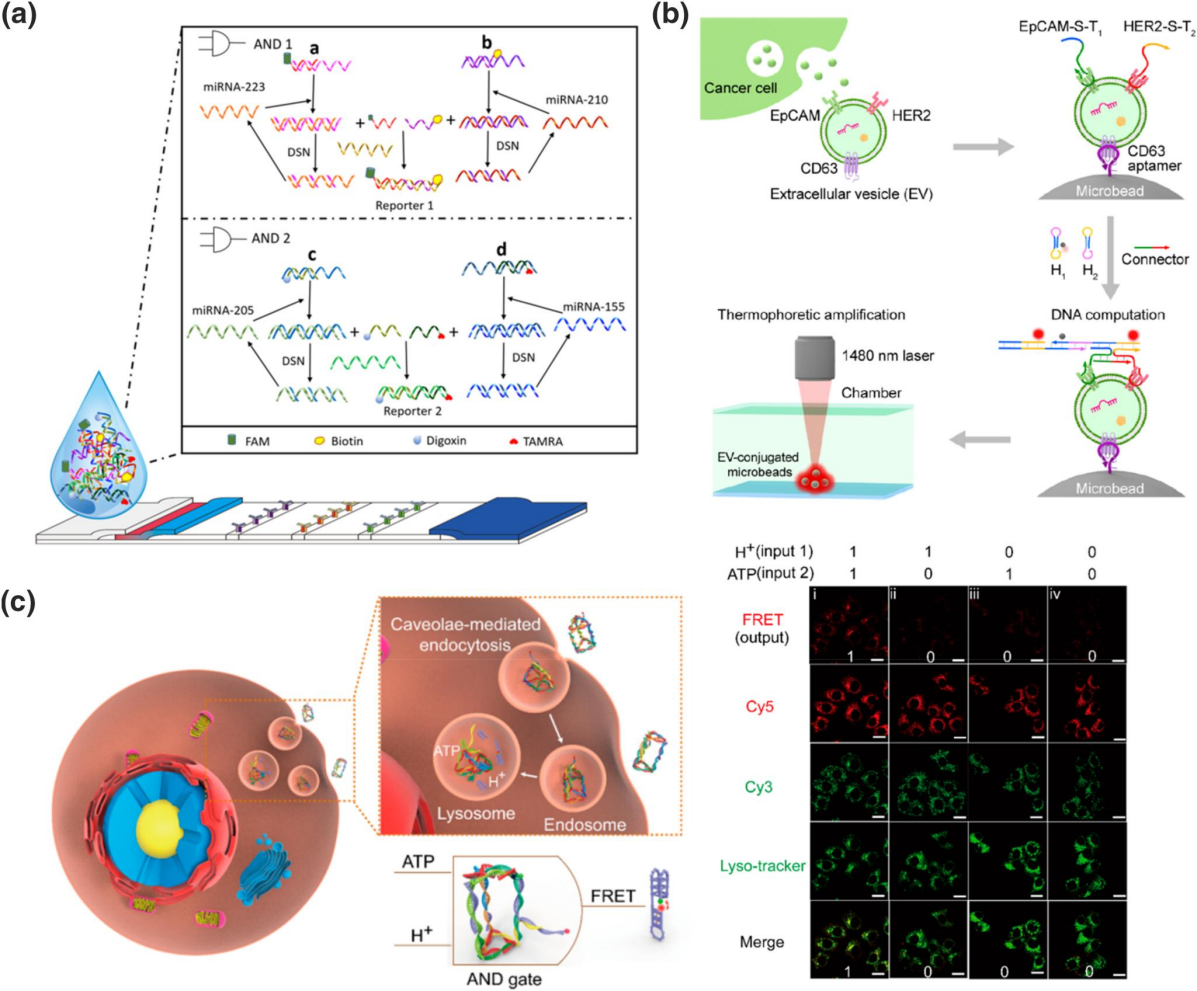
(a) A duplex‐specific nuclease‐mediated triple‐line lateral flow strip detection system with dual‐AND gate operations for detecting four lung cancer‐associated miRNAs. Reproduced with permission.^[82]^ Copyright © 2023, American Chemical Society. (b) Aptamer‐based DNA computation on the membrane of extracellular vesicles, the output signal was amplified by toehold‐activated hybridization chain reaction and thermophoresis. Reproduced with permission.^[83]^ Copyright © 2021, American Chemical Society. (c) DNA triangular prism with a three‐way branched edge containing the i‐motif and ATP‐binding aptamer sequences for lysosomal imaging (left panel). A decreased Förster resonance energy transfer signal reported by the DNA nanodevice when the cells were treated with chloroquine and oligomycin (right panel). Reproduced with permission.^[84]^ Copyright © 2019, American Chemical Society.

MicroRNAs (miRNAs) are single‐stranded non‐coding RNAs that play a crucial role in gene regulation.[^76^, ^77^, ^78^] However, elevated levels of miRNAs have been associated with various diseases, making them valuable biomarkers for disease diagnostics. The cell‐type‐specific expression patterns of miRNAs make them particularly useful for accurately distinguishing cancer types and stages of development. To address this, Yue et al. integrated molecular computation into a DNA‐programmed nanoparticle network to detect miRNA‐21 and miRNA‐122 within living cells.^[79]^ Their network consisted of GNPs functionalized with two DNAs (DNA 1 and DNA 2), a hetero‐bivalent DNA‐functionalized quantum dot (DNA3‐QD), and linker strands (Linker 1 and Linker 2). When both intracellular miRNA‐21 and miRNA‐122 were present, they displaced DNA3‐QD, leading to hybridization with Linkers 1 and 2. This complete disassembly of the network structure separated the quantum dots from the GNPs. Upon excitation, the quantum dot transitioned from a quenched to a fluorescent state, producing a TRUE output signal. In another study, Gong et al. developed miRNA‐initiated DNA circuits based on a HCR.^[80]^ Their DNA computation platform comprised sensing and processing modules. The sensing module involved the hybridization of designed metastable DNA hairpins with target miRNAs, releasing the initiator sequence. This triggered an autonomous and successive hybridization of other HCR hairpin reactants in the processing module. As a result, a selectively amplified FRET signal was detected. This simple working principle allowed the implementation of binary logic gates (OR, AND, INHIBIT, and XOR) and advanced concatenated logic circuits (XOR‐AND, XOR‐INHIBIT, and XOR‐OR) for miRNA detection in multiple cell lines. Chu's team employed a DNAzyme‐based approach to perform YES, OR, and AND logic operations in miRNA imaging.^[81]^ They utilized copper (II) ion‐doped zeolitic imidazolate framework‐8 nanoparticles (Cu/ZIF‐8 NPs) carrying immobilized DNAzyme logic gates on their surface. After cellular internalization, the DNAzyme logic gates were released in response to the acidic environment of endo‐lysosomes. The DNAzyme substrate region was initially blocked by a cDNA strand. When miRNA‐21, miRNA‐155, or both were present, a TMSD reaction occurred, freeing the substrate region. This activation led to Cu^2+^‐DNAzyme cleavage and the subsequent release of the Cy5‐containing DNA fragment. Furthermore, Zhou et al. developed a point‐of‐care (POC) testing system based on triple‐line lateral flow strips for the multi‐detection of lung cancer‐associated miRNAs (Figure 6a).^[82]^ Their system combined dual‐AND gate operations with duplex‐specific nuclease (DSN)‐mediated signal amplification to simultaneously detect four miRNAs (miRNA‐223, miRNA‐210, miRNA‐205, and miRNA‐155). They designed four fully cDNA strands (C‐DNA‐223/210/205/155) that were partially hybridized with complementary short RNAs (S‐RNA‐223/210/205/155). In AND gate 1, the presence of miRNA‐223 displaced the FAM‐modified S‐RNA‐223, forming a complex with C‐DNA‐223. Similar reaction occurred with miRNA‐210, releasing the biotin‐modified S‐RNA‐210. The DSN digested the DNA in the DNA/RNA duplex, releasing the captured miRNAs. The free miRNAs reacted with another C‐DNA, releasing more S‐RNAs. The released S‐RNA‐223 and S‐RNA‐210 hybridized with a long complementary strand, forming a reporter. The FAM of the reporter was immobilized on the tested line of a lateral flow strip through binding to anti‐FAM antibody, while the streptavidin‐labeled GNPs reacted with the biotin of the reporter, accumulating at the tested line for visual detection. Similar principle was applied to AND gate 2, using miRNA‐205 and miRNA‐155 as inputs, with digoxin and TAMAR replacing FAM and biotin, respectively. This sensing platform enabled rapid screening of miRNAs in clinical serum samples with 100% accuracy and a detection limit as low as 26.51 fM.

In addition to miRNAs, protein receptors on cell membranes serve as biomarkers for identifying diseased cells. DNA aptamers, generated through SELEX, show promising potential in recognizing unique receptors on the disease cell membrane. Tan's research team engineered an aptamer‐based DNA nanomachine that could recognize specific cancer cells using Boolean logic operations.^[85]^ The nanomachine consisted of a DNA triangular prism with two recognition toes (aptamers sgc8c and sgc4f) targeting human acute lymphoblastic leukemia cells (CCRF‐CEM), and a reporter toe. The recognition toes were initially blocked by cDNA. When both aptamers bound to their target receptors, aptamer/target conjugates were formed, releasing the cDNAs. The freed cDNAs underwent a strand displacement reaction with the reporter toe, restoring its fluorescence signal. This ON signal was observed only in CCRF‐CEM cells treated with the nanomachine, demonstrating its cellular specificity compared to control Ramos cells. Feng et al. also developed an aptamer‐based logic gate‐responsive DNA nanomachine for cell type recognition, utilizing RCA for signal amplification.^[55]^ Their design involved a ternary DNA complex formed by hybridizing a circular DNA template (CT) with a primer aptamer (PA) and a blocked aptamer (BA). Primer aptamer bound to the epithelial cell adhesion molecule (EpCAM) receptor, while BA targeted the mucin 1 (MUC1) receptor. The DNA ternary complex initially anchored itself to the cell membrane through PA‐EpCAM recognition. In the presence of MUC1 receptors, BA disassembled and complexed with the receptors, exposing the 3′ end of PA for nucleolytic digestion. The addition of phi29 DNA polymerase triggered the RCA process of the CT‐PA complex, generating a long ssDNA. A FAM‐labeled DNA oligonucleotide hybridized with the RCA products, producing a fluorescence signal specific to the target cells. This DNA ternary nanomachine functioned only when both EpCAM and MUC1 receptors were present, allowing precise differentiation of the breast adenocarcinoma cell line MCF‐7 in mixed cell samples.

Logic aptamer‐based diagnosis extends beyond membrane detection of cells and can also be applied to EVs. Li et al. developed a logic system using dual aptamers for the specific and sensitive identification of tumor‐derived EVs secreted by breast cancer (BC) cells (Figure 6b).^[83]^ The EVs were initially immobilized on microbeads coated with CD63 aptamers. Two aptamers targeting BC‐associated proteins on the cell membrane, EpCAM and human epidermal growth factor receptor 2 (HER2), were introduced. This was followed by a toehold‐activated HCR and thermophoresis to amplify the output fluorescence signal. This method successfully differentiated the BT‐474 cell line, which had high expression of both HER2 and EpCAM proteins, from two other breast cancer cells (MCF‐7 and UACC) and a human mammary epithelial cell line (MCF‐10A). Furthermore, their logic platform demonstrated the ability to discriminate between BC patients and healthy donors in clinical samples with a high accuracy of 97%.

The subcellular detection is a fascinating area of research, as each organelle plays a crucial role in maintaining diverse cellular functions. Dysfunctions within organelles can contribute to or be associated with a wide range of diseases.[^86^, ^87^] Recognizing this significance, DNA nanoprobes have emerged as valuable tools for organelle sensing. One intriguing component is the i‐motif, a cytosine‐rich sequence that can undergo dynamic structural switching to a quadruplex structure in response to acidic environments. Leveraging this property, Du et al. integrated the i‐motif and ATP‐binding aptamer into a framework nucleic acid (FNA) to develop a logic‐gated DNA nanodevice for subcellular imaging (Figure 6c).^[84]^ They constructed a DNA triangular prism with a three‐way branched edge containing the i‐motif and ATP‐binding aptamer. This DNA logic device was activated by both endogenous protons and ATP molecules present in the lysosomes. The formation of the i‐motif and ATP‐aptamer complex led to the disassembly of the FNA structure, resulting in an enhanced FRET signal. By detecting changes in FRET signals, the DNA logic device demonstrated the capability to sense elevated lysosomal pH and ATP depletion induced by inhibitors such as chloroquine and oligomycin (OL), respectively. This holds potential for diagnosing lysosomal storage disorders and ischemia.

In another study, Chai et al. developed a DNA nanodevice capable of imaging both glutathione (GSH) and ATP within mitochondria.^[88]^ They designed a DNA duplex comprising an ATP aptamer sequence and a complementary strand connected by a disulfide bond. In the presence of GSH, the disulfide bond was reduced, allowing the ATP aptamer sequence to separate from the unstable duplex. When ATP molecules were present, the fluorescence was restored as the dye moved away from the quencher molecule upon disassembly. To ensure mitochondrial targeting, the DNA nanodevice was delivered using nanoparticles decorated with a triphenylphosphonium moiety. This approach enabled specific AND‐gated molecular imaging of ATP and GSH within cells and exerted control over multiplexed imaging activities in tumor‐bearing mice.

### 3.2 Drug delivery for cancer therapy

**FIGURE 7:**
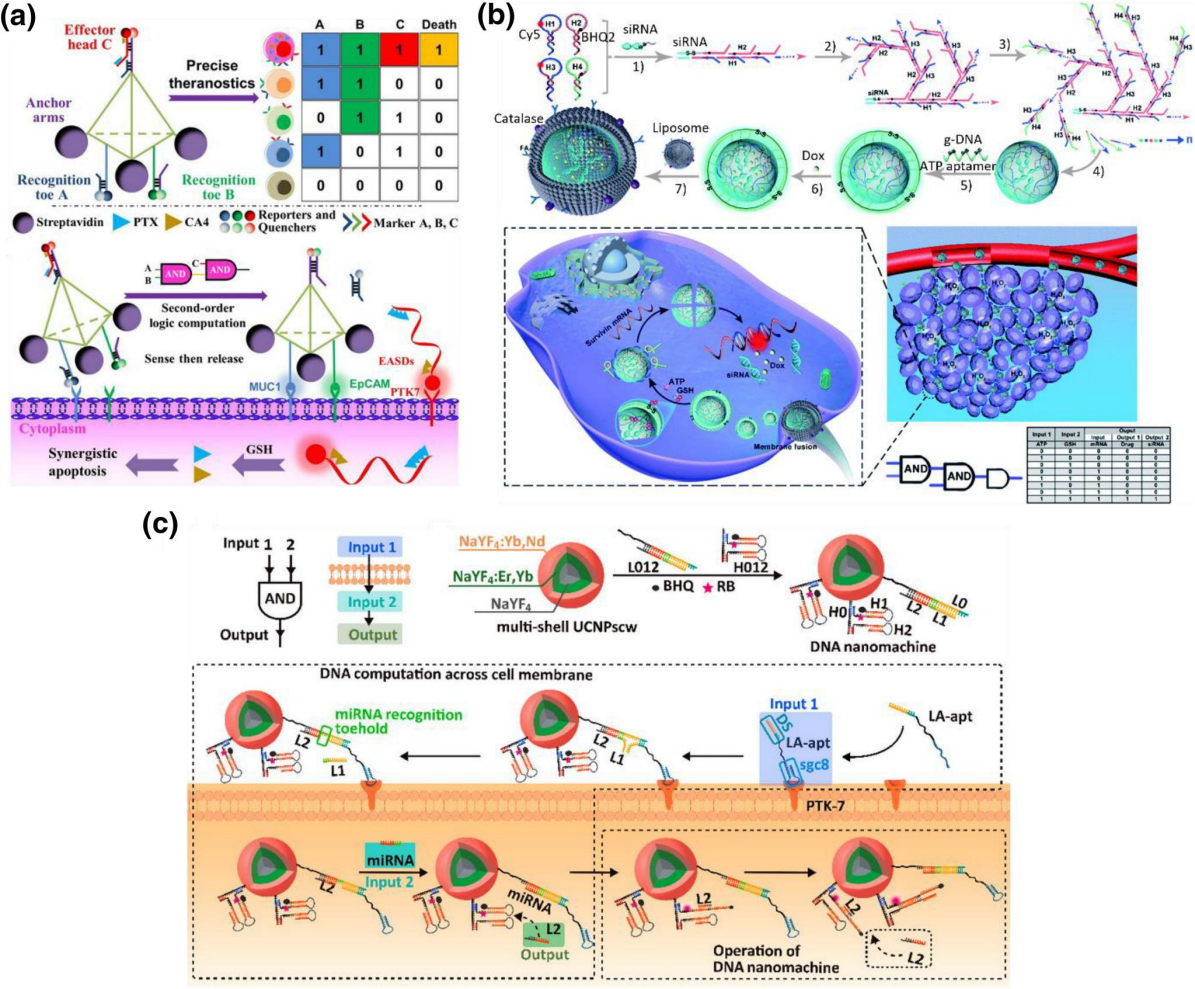
(a) DNA tetrahedron scaffold carried cascaded AND‐AND logic operation to deliver two synergistic drugs paclitaxel and combretastatin. Reproduced with permission.^[90]^ Copyright © 2021, John Wiley and Sons. (b) Specific intracellular release of siRNA and doxorubicin from a framework nucleic acid triggered by glutathione, adenosine triphosphate molecules and surviving mRNA. Reproduced with permission.^[91]^ Copyright © 2021, Royal Society of Chemistry. (c) Transmembrane logic computation by DNA nanomachine fabricated on NaYF_4_:Gd upconversion nanoparticle for photodynamic therapy. Reproduced with permission.^[92]^ Copyright © 2021, American Chemical Society.

DNA logic operations play a crucial role in achieving precise cancer therapy. Ouyang and colleagues developed a DNA‐based precision‐guided missile (D‐PGM) as a DNA logic vehicle.^[89]^ The D‐PGM consisted of a warhead (WH) and a guidance/control (GC) system. The GC section was assembled using three DNA strands that were partially complementary to three different aptamers (Sgc8, Sgc4f, and TC01), while the WH part was a tubular origami structure designed to carry doxorubicin (DOX). By pre‐incubating the aptamers Sgc8, Sgc4f, and TC01 with the cells, the target receptors were bound, sequentially disassembling the GC system and promoting the cellular internalization of D‐PGM. The release of DOX from D‐PGM was triggered by the intracellular acidic environment and enzymatic degradation of D‐PGM. The GC system in D‐PGM functioned as a three‐concatenated AND logic system, enabling targeted drug delivery with high cell‐type recognition. In another study, Zhang and Tan's group constructed a second‐order DNA logic‐gated nanorobot for the selective release of synergistic drugs based on multi‐aptamer‐mediated sensing (Figure 7a).^[90]^ The tetrahedral DNA scaffold was equipped with streptavidin for anchoring cell membranes and three aptamers for sensing three expressed markers. Two recognition aptamers at the robotic toes of the DNA nanorobot, designed to sense MUC1 and EpCAM markers, were initially blocked by complementary strands. When the DNA nanorobot targeted both markers, the removal of complementary strands activated the release of effector aptamer‐tethered synergistic drugs through a TMSD reaction. The effector aptamer, conjugated with combretastatin and paclitaxel, could be internalized by cells after binding to the PTK7 receptor. This strategy employed an AND‐AND logic operation, where the second gate could only be accessed after triggering the first AND gate. The designed DNA nanorobot exhibited high selectivity in drug delivery to cells with high expression of the three markers, resulting in significantly enhanced therapeutic effects.

The concept of logic operations can also be applied in gene therapy. Ma and colleagues combined gene therapy and chemotherapy in a triggered drug release system using dual miRNAs as inputs.^[93]^ They assembled two pairs of DNA‐RNA duplexes on the surface of DNA‐functionalized GNPs. Doxorubicin (DOX) was intercalated into the base pairs of nucleic acid hybrids. When miRNA‐21 and miRNA‐10b were present as inputs, they catalyzed the dissociation of DNA‐RNA duplexes through a strand displacement reaction. This led to the generation of siRNA to suppress the Bcl‐2 protein level and the release of DOX. Na's team utilized two intracellular biomarkers, GSH and ATP, as inputs to trigger the release of siRNA and DOX in a logical manner for cancer cells.^[91]^ They constructed a spherical FNA through the HCR of four hairpin DNA monomers, using siRNA as an initiator (Figure 7b). The FNA core was covered by a g‐DNA consisting of S‐S linkages and ATP‐aptamers, which responded to GSH and ATP molecules. DOX was loaded into the FNA particles for chemotherapy. The high expression of GSH and ATP in cancer cells cleaved the g‐DNA, exposing the FNA core. Two of the hairpin monomers, H1 and H3, had the ability to hybridize with survivin mRNA, causing the core structure to collapse and release siRNA and DOX. The released siRNA silenced the gene expression of the protein polo‐like kinase 1, and in combination with DOX, their cascaded‐logical‐release system successfully enhanced the synergistic antitumor effect in tumor‐bearing mice.

Researchers are actively exploring the integration of DNA logical computation strategies into photodynamic therapy (PDT). In a study by Zhang et al., a DNA nanomachine was developed on NaYF4:Gd upconversion nanoparticles (UCNPs) to perform transmembrane AND gate operations (Figure 7c).^[92]^ The first input consisted of an 18‐base DNA segment conjugated with the sgc8 aptamer (LA‐apt), which could bind to PTK‐7 receptors on the cell membrane's surface. The 18‐base segment interacted with the DNA nanomachine on UCNPs, triggering a strand displacement reaction to create a miRNA recognition toehold. The presence of LA‐apt facilitated the endocytosis of the UCNP system by anchoring the DNA nanomachine to the cell membrane of targeted cancer cells. Upon endocytosis, intracellular miRNA‐21 served as the second input, hybridizing with the miRNA recognition toehold and releasing a leaving strand (L2). L2 then hybridized with a DNA hairpin labeled with the photosensitizer Rose Bengal (RB), causing the hairpin structure to unfold and activate RB for the generation of reactive oxygen species (ROS) under green light emitted by UCNPs upon 808 nm light irradiation. This strategy ensured the specific uptake of the UCNP system by cancer cells in complex solid tumor microenvironments, enabling precise PDT while minimizing toxicity to non‐target cells. In a recent study, Li et al. designed a DNA logic circuit for photodynamic therapy using dual aptamers as inputs.^[94]^ They introduced two DNA hairpin structures, each with aptamers Sgc‐8c and Sgc‐4f, to target cancer cells. The two hairpins could anchor onto the cell membrane by binding to specific target receptors, triggering a HCR. Additionally, a DNA hairpin with a preloaded G4 containing the photosensitizer 5,10,15,20‐tetrakis‐(N‐methyl‐4‐pyridyl) porphyrin (TMPyP4) was immobilized on cancer cells with a specific receptor expression. Under blue light irradiation, cytotoxic ROS were generated, selectively killing the specific type of cancer cells. This dual aptamer‐based logic operation enabled the recognition of specific cell types for targeted photodynamic therapy.

### 3.3 Monitoring and modulation of cellular activities

**FIGURE 8:**
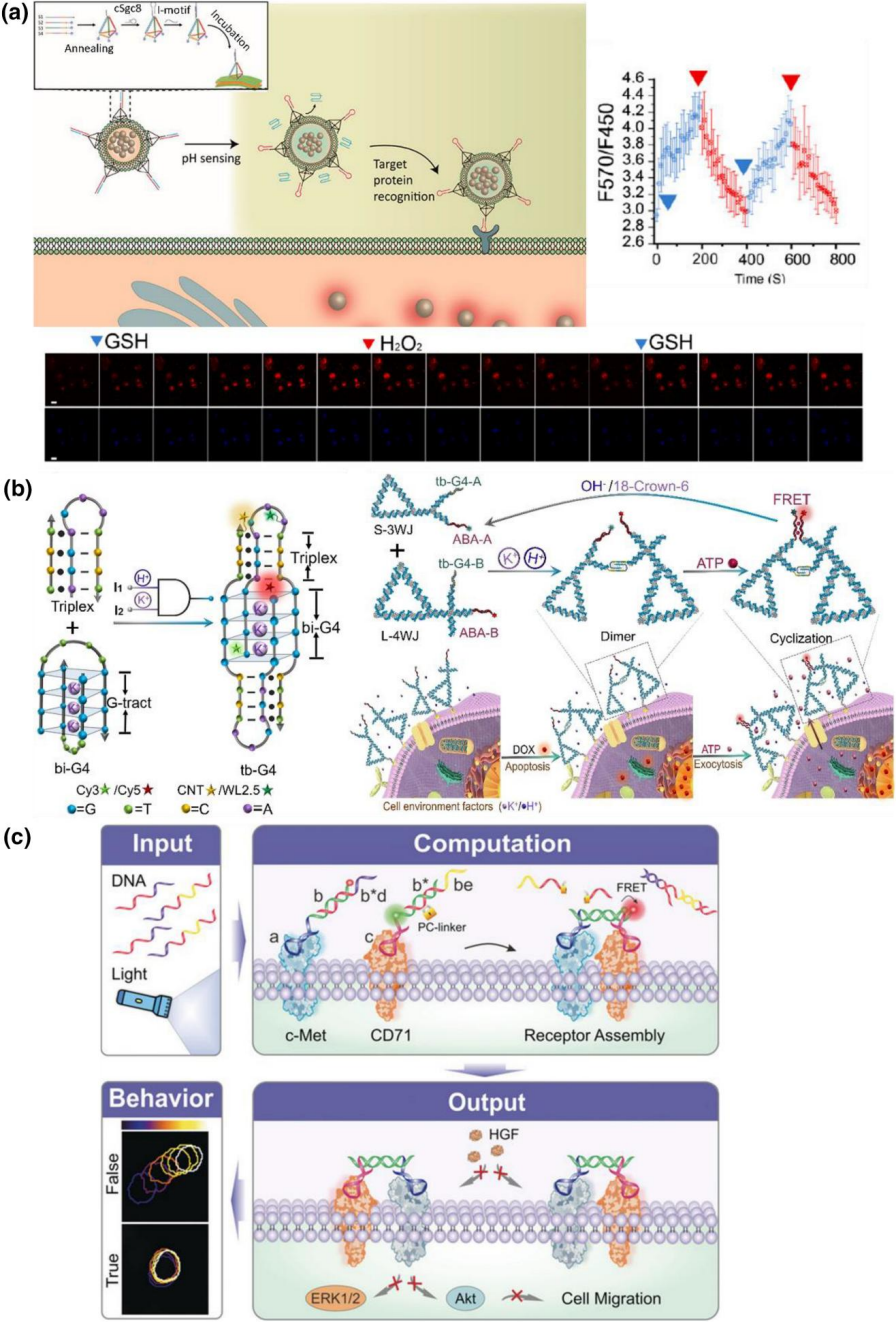
(a) A DNA logic‐gated nanovesicle encapsulated with gold carbon dots for examining the change of redox status by analysis of ratiometric fluorescence during imaging. Reproduced with permission.^[95]^ Copyright © 2021, American Chemical Society. (b) An apoptosis detection platform built by two DNA nanotriangles tethered with triplex‐boosted G‐quadruplex and split adenosine triphosphate aptamer. ATP molecules released by DOX‐induced apoptotic cells were captured by the dimerized DNA nanotriangles to show the early apoptosis stages of cells. Reproduced with permission.^[96]^ Copyright © 2021, John Wiley and Sons. (c) Aptamer‐based DNA logic system for modulation of signal transduction. Dimerization of c‐Met and CD71 receptors was controlled by DNA or light‐input guided DNA assembly, resulting in an inhibition of the c‐Met/hepatocyte growth factor signal pathway. Reproduced with permission.^[97]^ Copyright © 2019, John Wiley and Sons.

Cellular recognition through DNA computation not only enhances the precision of cancer diagnosis and drug efficacy but also enables the monitoring of dynamic cellular activities. Tan et al. developed a DNA‐based logic‐gated nanovesicle incorporated with gold carbon dots (GCDs) to monitor the redox status of cancer cells (Figure 8a).^[95]^ Cell membrane nanovesicles derived from living cells were modified with cholesterol‐conjugated DNA tetrahedral scaffolds. The vertex of the DNA tetrahedron was attached to a duplex consisting of the sgc‐8 aptamer and i‐motif sequences. When exposed to an acidic environment, the i‐motif sequence separated and formed a parallel‐stranded structure, restoring the recognition capability of the sgc‐8 aptamer to the PTK7 receptor. Utilizing the DNA logic gate, this system directed the delivery of GCDs encapsulated within the nanovesicles to target cells, enabling the monitoring of intracellular redox status fluctuations. The system detected changes in the ratiometric fluorescence (570 nm/450 nm) of GCDs upon the alternate addition of GSH and hydrogen peroxide at different time points.

In the detection of telomerase activities, Zhu's group demonstrated the application of DNA computation.^[98]^ They constructed a cascade DNA‐based YES‐AND logic circuit that responded to the intracellular telomerase activity. Telomerase served as the first input, elongating a telomerase substrate (TS) primer to generate an extended TS + nR strand. The TS + nR strand displaced a partially hybridized DNA strand in a duplex containing a fluorophore‐quencher pair, leaving a new toehold domain. Another oligonucleotide, referred to as Input B, acted as the second input for the AND gate computation, triggering the strand displacement reaction. The output signal was reported in terms of fluorescence intensity. his DNA computation device enabled the imaging and quantification of telomerase activity in different cancer cell lines, facilitating the distinction between cancerous and normal cells. Additionally, the device responded to changes in telomerase activity after treatment with different concentrations of epigallocatechin‐3‐gallate (EGCG), allowing the quantification of the suppressed enzymatic activity by measuring the decrease in fluorescence intensity. These findings contribute to the investigation of the association between cancer and telomerase and the development of telomerase inhibitors based on DNA computation techniques.

Apoptosis, another intracellular state, can also be detected through DNA computation. Zheng et al. designed a FNA platform with an AND gate function to monitor cell apoptosis (Figure 8b).^[96]^ They created two DNA nanotriangles with three‐way and four‐way junctions, tethered with triplex‐boosted G4 and split ATP aptamer. The acidic microenvironment and presence of potassium ions promoted the formation of DNA triplexes and G‐quadruplexes, anchoring the two proximate DNA nanotriangles to the cell membrane. adenosine triphosphate molecules secreted from cancer cells during DOX‐induced apoptosis were captured by the split ATP aptamer, resulting in a FRET signal. Through real‐time probing, the FNA nanoplatform generated an observable signal within 0.5 h after the introduction of DOX to cancer cells, without detecting cytotoxicity through the MTT assay. These results indicated that the designed nanoplatform was capable of detecting apoptosis at an early stage and evaluating therapeutic efficacy by sensing released ATP.

DNA‐based computation offers a non‐genetic and programmable approach for modulating cellular signal transduction. Yang's team developed a set of logic gates (YES, OR, AND, NOR, and AND/OR) based on aptamer recognition and DNA assembly to regulate the mesenchymal epithelial transition (c‐Met)‐hepatocyte growth factor (HGF) signaling pathways (Figure 8c).^[97]^ They engineered two aptamers with partially complementary double‐stranded structures to capture c‐Met and CD71 receptors. Through designated inputs of DNA oligonucleotides or light irradiation, the blocker strand was removed via a strand‐displacement reaction. The assembly of the two aptamer‐containing strands brought c‐Met and CD71 receptors in close proximity, inhibiting the interactions between c‐Met and HGF due to strong steric hindrance. Consequently, the cellular migration ability was inhibited, as demonstrated in a wound‐healing assay. Similar concept was demonstrated in the recent work by Wu and colleagues. They designed Strand M, which contained an aptamer region that could bind to the c‐Met receptor, and a sequence complementary to Strand A, where Strand A was an ATP aptamer. Strand T included a CD71‐targeted aptamer, but it was initially blocked by Strand I, an i‐motif DNA strand. Under normal conditions, the two duplexes anchored on c‐Met and CD71 proteins did not induce receptor dimerization. However, in a tumor‐like microenvironment, Strand A and Strand I were separated from the duplexes in response to high ATP expression and an acidic environment. This triggered the assembly of Strand M and Strand T, leading to the proximal assembly of c‐Met and CD71. The dimerization of c‐Met with CD71 proteins interfered with the c‐Met/HGF signaling pathway, inducing cytoskeletal reorganization and inhibiting cancer cell mobility.^[99]^

### 3.4 Logic operations in synthetic biology

Biological computation is one of the compelling research topics in synthetic biology that establishes biomolecular‐based machines to perform predefined functions in a controllable and logical manner. Logic gates are critical to machinery operations and computation, making biomolecular‐based logic gates necessary in synthetic biology. Over the past years, many biological gates have been created by comprising various biomolecules including DNA, RNA, proteins and signaling molecules.[^100^, ^101^] Among the biomolecular candidates, DNA has the merits of high programmability and structural predictability, so it is capable of processing complex logic operations,[^102^, ^103^, ^104^] making it favorable to be adopted in developing biological gates. Chen and co‐workers utilized DNA strand displacement to engineer synthetic protein switches to control the proximity of two proteins dynamically and logically.^[105]^ The protein‐DNA complexes prepared were hybridized with partially complementary sequences, and the assembly of proteins was controlled by the input of DNA strands to trigger TMSD reactions. Their reported strategy enabled the DNA‐guided assembly of split enzyme yeast cytosine deaminase (yCD) in response to the inputs of microRNAs miR‐21 and miR‐122. The reconstitution of split yCD activated the prodrug 5‐fluorocytosine (5‐FC) to 5‐fluorouracil (5‐FU) to inhibit DNA synthesis. This synthetic DNA‐based computing platform could regulate the dynamic protein assembly in a logic‐guide manner. Joesaar et al. developed semipermeable proteinosome‐based protocells to encapsulate DNA strand displacement circuits.^[106]^ The internalized DNA circuits could encode and decode input messages by single‐strand DNA strands based on the TMSD reactions, allowing a signaling cascade between individual protocells. Their idea demonstrated the potentiality of synthetic cell‐cell communication systems in biologically associated conditions using DNA‐based devices. The team of Wilson introduced a programmed gene regulation in *Escherichia coli* (*E. coli*) using engineered transcription factors and complementary genetic architectures.^[107]^ Taking the principle of transcriptional regulation by lactose repressor, they adapted non‐natural transcription factors, DNA recognition units and DNA promoters in designing the fundamental logic gates including AND, OR, NOT, NOR and two non‐canonical half‐AND operations to modulate the gene expression. They also built complex logic circuits by employing parallel and series genetic architectures. The readout of output signals was reported as the expression of green fluorescence protein (GFP), where the DNA operator was located upstream of a GFP reporter. In contrast to the fundamental capability of DNA to form double helixes by two complementary strands, Nikitin constructed a DNA logic circuit based on non‐complementary strands.^[108]^ Short ssDNA oligonucleotides with a weak interaction with each other were utilized to build multiple‐input AND, OR and NAND gates and a square‐root circuit for 4‐bit inputs. Furthermore, the author regulated the expression of Firefly Luciferase mRNA by the DNA logic circuit based on the strand commutation mechanism.

## 4 CONCLUSION AND FUTURE PERSPECTIVES

The field of stimuli‐responsive DNA‐based logic gates presents a fascinating avenue for interdisciplinary research, offering immense potential in diverse domains. By leveraging the programmability and responsiveness of DNA molecules, researchers are pushing the boundaries of computation and engineering at the nanoscale.

Despite the progress made, there are still several challenges that need to be addressed for the continued development and application of stimuli‐responsive DNA‐based logic gates. Firstly, the design and optimization of aptamers with high affinity and specificity for target receptors remain crucial. Improving the binding efficiency and selectivity of the aptamers will enhance the precision and effectiveness of the logic gate systems. Another challenge lies in the stability and durability of the DNA‐based constructs within cellular environments. Ensuring the stability of the logic gate components, especially in the presence of nucleases and other degrading agents, is essential for their long‐term functionality and applicability in biological systems. Furthermore, the integration of stimuli‐responsive DNA‐based logic gates with complex cellular networks poses a significant challenge. Cellular signaling pathways are highly interconnected and dynamic, requiring careful consideration of the interactions and crosstalk between different pathways. Developing strategies to integrate and interface DNA‐based logic gates with existing cellular networks will be crucial for their successful implementation in complex biological systems. Future perspectives for stimuli‐responsive DNA‐based logic gates involve expanding their applications beyond the simple modulation of cellular functions. These gates hold potential for advanced therapeutic interventions, such as targeted drug delivery, precision medicine, and tissue engineering. Additionally, integrating DNA‐based logic gates with other emerging technologies, such as nanotechnology and synthetic biology, could enable the development of highly sophisticated and adaptable systems for precise control of cellular behavior.

## CONFLICT OF INTEREST STATEMENT

The authors declare no conflicts of interest.

## Data Availability

Data sharing is not applicable to this article as no new data were created or analyzed in this study.
